# Successful treatment of oral Crohn's disease by anti-TNF-alpha dose escalation - a case report

**DOI:** 10.1186/s12876-018-0818-7

**Published:** 2018-06-18

**Authors:** Arne Bokemeyer, Nicolas Tentrop, Peter J. Barth, Frank Lenze, Karin Hengst, Johannes Kleinheinz, Dominik Bettenworth

**Affiliations:** 1 Department of Medicine B for Gastroenterology and Hepatology, University Hospital Münster, Albert-Schweitzer-Campus 1, D-48149 Münster, Germany; 2 Department of Cranio-Maxillofacial Surgery, University Hospital Münster, Albert-Schweitzer-Campus 1, D-48149 Münster, Germany; 3 Gerhard-Domagk-Institute of Pathology, University Hospital Münster, Albert-Schweitzer-Campus 1, D-48149 Münster, Germany

**Keywords:** Inflammatory bowel disease, Crohn’s disease, Immunosuppressive therapy, TNF-alpha-therapy

## Abstract

**Background:**

Crohn’s Disease (CD) is typically characterized by abdominal symptoms, however, besides gastrointestinal symptoms, CD patients may suffer from extraintestinal manifestations which are far less common and medical treatment can be challenging.

**Case presentation:**

We report about a 34-year-old Crohn’s Disease (CD) patient in clinical remission under adalimumab therapy who presented in the clinic for Cranio-Maxillo Surgery due to severe pain in the mandibular area. Ulcerative lesions of the buccal-side mucosa of the right mandible were detected. To rule out malignancy, a biopsy was obtained and revealed ulcerative stomatitis with noncaseating granulomas consistent with oral CD. Shortening the adalimumab administration interval to weekly injections resulted in a complete healing of the oral CD lesions without residual inflammation.

**Conclusion:**

The case presented here demonstrates that gastroenterologists should evaluate and consider oral CD lesions as a possible marker of disease activity in patients despite having quiescent intestinal CD.

## Background

Inflammatory bowel disease (IBD), including Crohn’s disease (CD), are frequent inflammatory disorders of the gastrointestinal tract [1]. Patients with a flare-up of disease frequently present with inflammation-associated symptoms like abdominal pain, diarrhea and fever [1]. Besides frequent gastrointestinal symptoms, extraintestinal manifestations of CD are far less common in these patients and medical treatment can be challenging.

## Case presentation

**Fig. 1:**
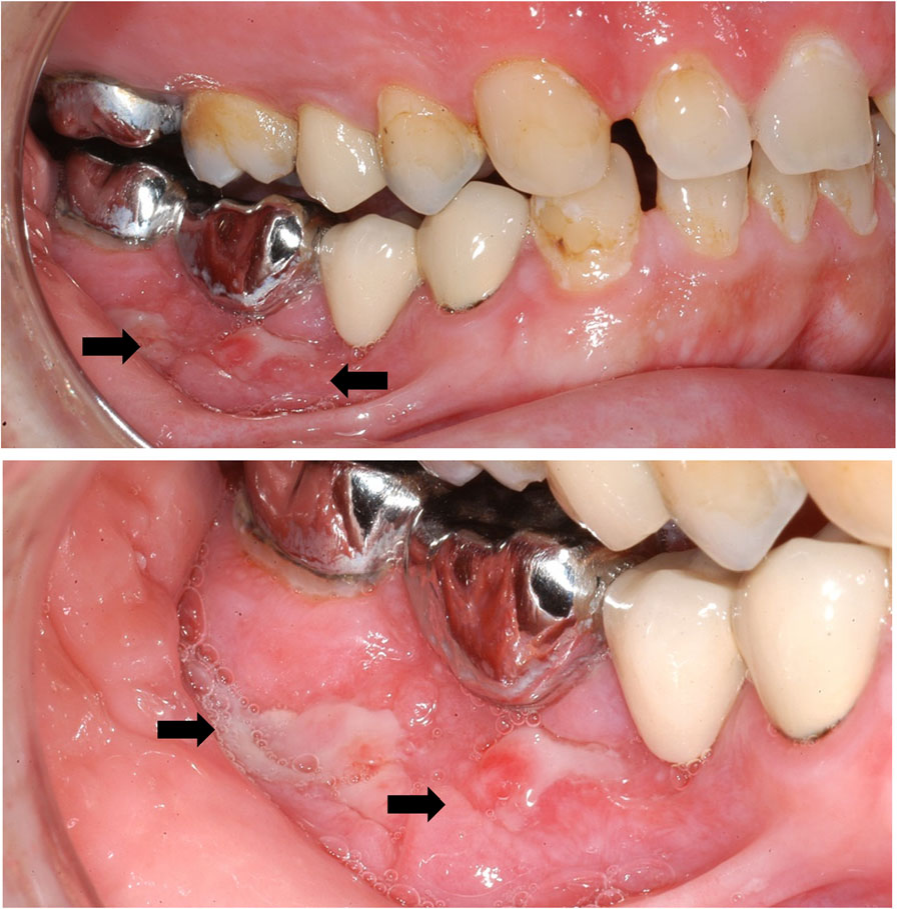
Examination of the oral cavity. The examination of the oral cavity detected ulcerative lesions of the buccal-side mucosa of the right mandible

**Fig. 2:**
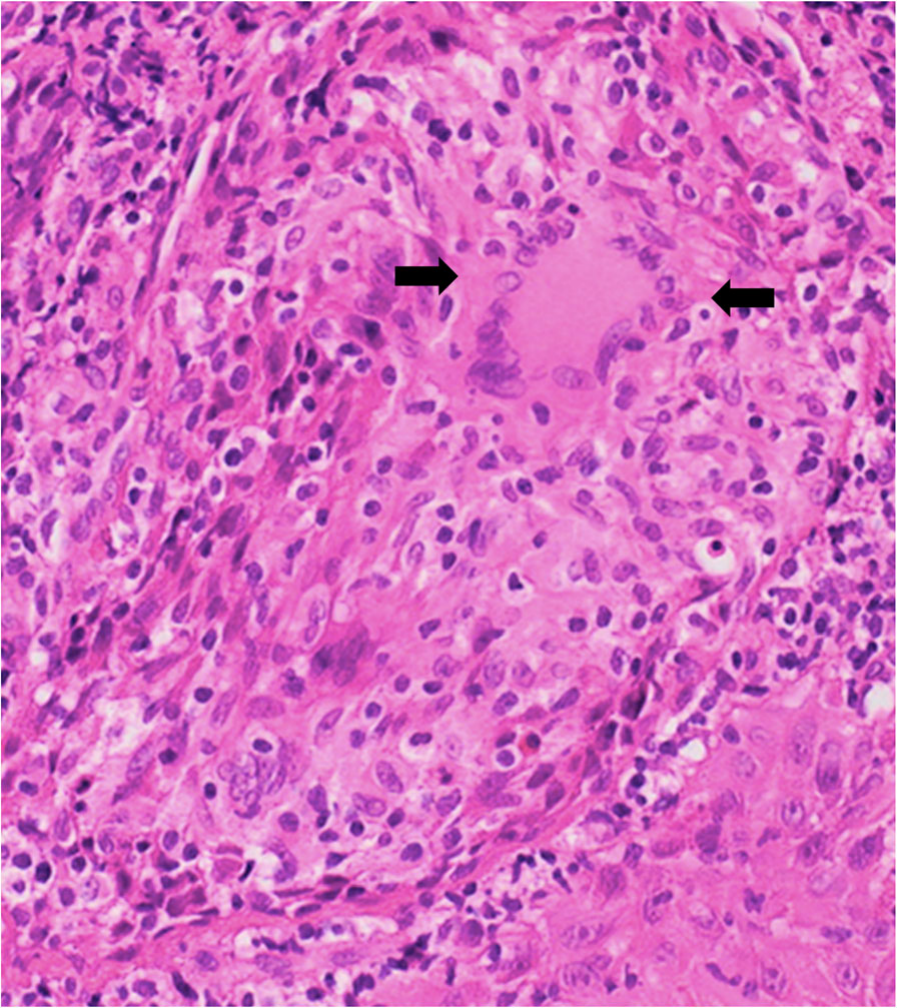
Histological evaluation of the oral biopsy. The histopathological evaluation of the oral biopsy revealed an ulcerative stomatitis with noncaseating granulomas consistent with oral CD

**Fig. 3:**
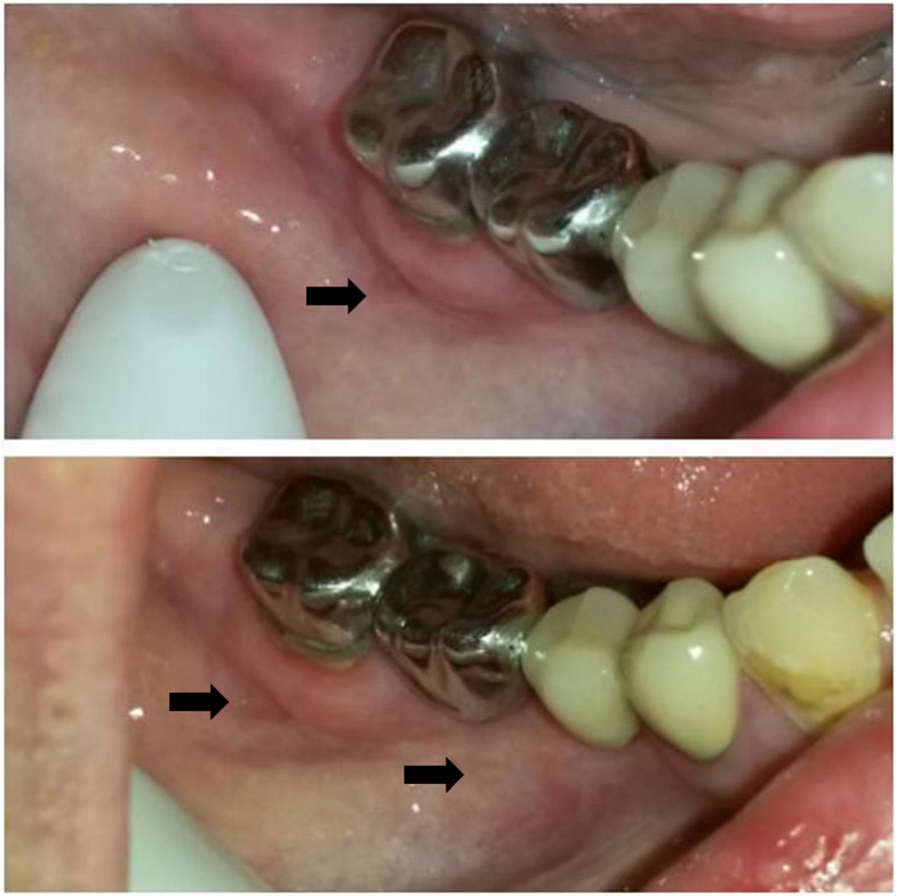
Follow-up examination after 10 weeks. A follow-up examination after 10 weeks confirmed a complete healing of the oral CD lesions

A 34-year-old man with a 15-year history of Crohn’s Disease (CD) was admitted to our hospital due to abdominal pain, non-bloody diarrhea and weight loss. Physical examination demonstrated moderate abdominal tenderness with an abdominal mass in the right lower quadrant. Laboratory findings revealed a significantly elevated C-reactive protein (CRP 7.5 mg/dl). Colonoscopy with ulcerations localized at the Bauhin’s valve and histological examination of obtained mucosal biopsies were suggestive for active CD. As endoscopic intubation of the terminal ileum was not possible, MR enteroclysis was performed and indicative of a predominant inflammatory, short-segment stenosis of the terminal ileum. Given the acute disease flare and the stricturing phenotype, medical treatment was switched from prednisolone and azathioprine to the anti-tumor-necrosis-factor (TNF)-alpha antibody adalimumab. Twelve weeks after induction of adalimumab therapy, clinical remission was achieved and CRP level returned to normal. Another four months later, clinical remission was still maintained and laboratory inflammation markers remained low, but the patient presented in the clinic for Cranio-Maxillo Surgery due to severe pain in the mandibular area. Examination of the oral cavity detected ulcerative lesions of the buccal-side mucosa of the right mandible (Fig. 1). To rule out malignancy, a biopsy of the oral lesions was obtained and revealed ulcerative stomatitis with noncaseating granulomas consistent with oral CD (Fig. 2). Intensification of immunosuppressive therapy was initiated by shortening the adalimumab administration interval to weekly administration. A follow-up examination after 10 weeks confirmed complete healing of the oral CD lesion (Fig. 3). During a follow-up period of 12 months, no signs of active CD became evident under continued therapy.

## Discussion and conclusions

While CD commonly manifests in the intestine of affected patients, oral lesions like aphthous ulcers or stomatitis are rare and occur only in approximately 10% of patients [2]. A recently published systematic review on oral CD manifestations in pediatric patient cohorts indicates that oral lesions can develop coincidently with gastrointestinal inflammation or even precede and thus may represent the initial sign of another disease flare [3]. Medical treatment of these oral lesions can be challenging and published evidence on medical treatment efficacy for oral CD lesions is limited [4]. Besides a few case reports, a most recently published study by Vavricka et al. documents a response rate of 78% for anti-TNF treatment in 32 adult IBD patients with oral disease manifestations [5, 6]. Additionally, our case presented here, demonstrates that anti-TNF therapy intensification can also represent a successful treatment approach in CD patients with oral disease lesions. Therapeutic drug monitoring was not available at the time the patient was treated at our institution, but is nowadays widely spread and can facilitate clinical decision making in IBD patients with primary or secondary loss of response towards anti-TNF treatment.

Concluding, oral lesions are a rare manifestation of CD and gastroenterologists should consider these lesions as a possible marker of disease activity in patients despite having quiescent intestinal CD.
